# Cost of Venous Thromboembolic Disease in Patients with Lung Cancer: COSTECAT Study

**DOI:** 10.3390/ijerph18020394

**Published:** 2021-01-06

**Authors:** Ana Rosa Rubio-Salvador, Vicente Escudero-Vilaplana, José Antonio Marcos Rodríguez, Irene Mangues-Bafalluy, Beatriz Bernardez, Carlos García Collado, Roberto Collado-Borrell, María Dolores Alvarado Fernández, José Ignacio Chacón López-Muñiz, María Yébenes Cortés, Manuel Gómez Barrera, Miguel Ángel Calleja-Hernández

**Affiliations:** 1 Servicio de Farmacia, Complejo Hospitalario de Toledo, 45004 Toledo, Spain; 2 Servicio de Farmacia, Hospital General Universitario Gregorio Marañón, 28007 Madrid, Spain; vicente.escudero@salud.madrid.org (V.E.-V.); roberto.collado@salud.madrid.org (R.C.-B.); 3 Servicio de Farmacia, Hospital Universitario Virgen Macarena, 41009 Sevilla, Spain; jose.marcos.sspa@juntadeandalucia.es (J.A.M.R.); mdoalvarado@gmail.com (M.D.A.F.); mangel.calleja.sspa@juntadeandalucia.es (M.Á.C.-H.); 4 Servicio de Farmacia, Hospital Universitari Arnau de Vilanova, 25198 Lleida, Spain; imangues.lleida.ics@gencat.cat; 5 Servicio de Farmacia, Hospital Clínico Universitario de Santiago de Compostela, Santiago de Compostela, 15706 A Coruña, Spain; beatriz.bernardez.ferran@sergas.es; 6 Servicio de Farmacia. Hospital Universitario Virgen de las Nieves, 18014 Granada, Spain; carlosg.garcia.sspa@juntadeandalucia.es; 7 Servicio de Oncología Médica, Complejo Hospitalario de Toledo, 45007 Toledo, Spain; jignaciochacon@gmail.com; 8 Pharmacoeconomics & Outcomes Research Iberia, 28224 Madrid, Spain; myebenes@porib.com (M.Y.C.); mgomezbarrera@porib.com (M.G.B.)

**Keywords:** cancer, thromboembolic disease, LMWH, cost, economic impact

## Abstract

Background: Patients with lung cancer (LC) are at significantly higher risk of developing venous thromboembolism (VTE), which may lead to increased use of health resources and the cost of the disease management. The main aim of the study was to determine the cost of the management of VTE events in patients with LC treated with Low Molecular Weight Heparins (LMWH) in Spain. Methods: Costecat was an observational, ambispective pharmacoeconomic study. Patients with LC, with a first episode of VTE (symptomatic or incidental) in treatment with LMWH, were recruited from six third-level hospitals and followed up for six months. Sociodemographic, clinical and resource use variables of VTE-related implications and its treatment were collected. Direct healthcare costs and direct non-healthcare costs were recorded. Data collection was documented in an electronic case report. Costs (€2018) were estimated from the healthcare perspective. Statistical analysis was performed using the statistical program R 3.4.3 version (30 November 2017). Results: Forty-seven patients were included. Mean age was 65.4 years, 66.0% were male. The percentage of patients with LC who had metastatic disease was 78.7%. Twenty-three patients (48.9%) needed hospital admissions due to thromboembolic episode. Total average cost of patients with cancer associated VTE (CAT) was €10,969.6 per patient/semester. The hospitalizations represent 65.8% of total costs (7207.3 € SD 13,996.9 €), followed by LMWH therapy which represents 18.6% (2033.8 € SD:630.5 €). Conclusions: Venous thromboembolism episodes induce an economic impact on patients and healthcare systems. Direct healthcare costs are the major burden of the total cost, in which hospitalizations are the main drivers of cost.

## 1. Introduction

In a context of public and universalistic healthcare service structured, among all factors included in public healthcare expenditure (PHE), such as directly provided services (DPS), pharmaceutical care, general practitioner care, specialist medical care, privately delivered hospital care, other privately delivered medical services, and psychiatric support and rehabilitation, only DPS was related to the all-cause mortality rate (MR), representing the driving force of the system and a determinant of the population health [1].

Venous thromboembolism (VTE), including pulmonary embolism (PE) and deep vein thrombosis (DVT), constitutes a major health problem in developed countries. It has been estimated that VTE is responsible for more than 300,000 hospital admissions and 50,000 deaths each year in the United States. The pathogenesis of VTE is multifactorial, involving acquired risk factors, such as surgery, trauma, malignant disease, and genetic factors [2].

VTE represented 0.82% (0.69–0.92%) of all hospital discharges in Spain between 1999 and 2005. The rate of diagnoses for all hospital discharges in 2005 was 103/100,000 inhabitants, with an estimated number of total diagnoses in Spain (hospitalized or not) of 154/100,000 [3].

In cancer patients, VTE is one of the principal causes of morbidity and mortality [4,5,6]. Large population-based studies and disease registry surveys have revealed that around 20% of patients with VTE have active cancer and, in these patients, VTE is not only associated with shortened survival, but it also impacts quality of life [7].

Although the pathogenesis of thromboembolic events in cancer is likely multifactorial, chemotherapy and immobilization are considered two of the risk factors to develop thromboembolic events [5,6]. Cancer-associated VTE (CAT) occurs in 4–20% of cancer patients; with these with as elderly age at diagnosis, increase of cancer prevalence, advances in the diagnosis of incidental VTE, and anti-tumoral drugs with thrombotic properties being the factors most frequently associated. The main clinical practice guidelines recognize LMWH as the standard treatment of care for CAT. Treatment should start early after diagnosis with a median duration treatment of 3–6 months [8,9]. This specific group of patients presents high healthcare and non-healthcare resource utilization. Patients often suffer a higher number of hemorrhagic complications and VTE recurrences which may increase hospitalizations, hospital stays, specialist visits, prescription drugs, resources used for the diagnosis and management of VTE and other requirements [10]. Several studies have evaluated the burden of CAT especially the impact on hospitalization. However, most of these studies have been published in the United States or in other European countries, but not in Spain, so the authors consider that there is a need of a local study in order to show the costs of VTE.

Clinicians who treat CAT should be aware that this event is not merely a complication but has a broader impact on the patient’s quality of life [11]. Lung cancer (LC) is the most prevalent cancer worldwide with an estimated 1.6 million new cases per year and 1.38 million deaths. This type of cancer is associated with a high incidence of VTE. Surgery, chemotherapy and disease progression are considered risk factors to develop VTE [12]. Lung cancer is the third most common cancer diagnosed in Spain in 2018, the age-standardized incidence rate was 42.1 in males and 14.0 in females. [13].

To date, there are no published studies to report directly provided services, in terms of pharmaceutical care expenditure related to VTE in Spanish population of lung cancer patients. The knowledge of the economic impact of VTE in cancer patients would lead us to allocate adequate resources in primary prevention and early diagnosis and treatment measures to optimize the public healthcare expenditure in this group of population. The aim of this Study was to determine the cost of venous thromboembolic disease in patients with lung cancer in Spain.

## 2. Materials and Methods

COSTECAT was an observational, ambispective, pharmacoeconomic, multicentre study of outcomes and costs of the disease in the Spanish healthcare perspective. The study was carried out in six third level hospitals from Madrid, Catalonia, Andalusia, Galicia, and Castilla La Mancha Communities. The study was approved by the Andalusia Biomedical Research Ethics Coordination Committee (Register Number 2/Feb/2017-2100/2204) and notified to the Spanish Medicines Agency (14807/RG25745). The Spanish health system is a decentralized system with the health decisions transferred to the autonomous communities. The study comprises five different communities that represent areas of higher and lower population density and different access to health services, so they are considered representative of the country.

The recruitment period for patients in the COSTECAT study was established from October 2017 to April 2018. Each patient was monitored for six months from the date of recruitment. The study was conducted with patients who met the inclusion criteria: All of the participants were aged older than 18 years, with any stage of active LC according to the seventh edition staging system of American Joint Committee on Cancer, patients who suffered a first episode of a symptomatic or incidental VTE and were received treatment with LMWH. Life expectancy had to be ≥12 weeks. All patients signed an informed consent.

The data was obtained from patient medical records, directly form a personal interview during the baseline visit, and from a patient diary during the six months follow-up period. Data was collected in an electronic case report form. We collected sociodemographic (age, gender, weight, height), clinical (TNM staging system, Eastern Cooperative Oncology Group (ECOG) performance status on grading cancer, cancer treatment, chronic complications, bleeding history and risk factors, renal function impairment, history thromboembolic disease, LMWH treatment) and economic data related to direct cost associated with VTE (inpatient stays, emergency visit, healthcare specialist visits, clinical tests). Direct non-healthcare resources included formal social services, informal care, and adapted medical transport and physical adaptations. Patients included in the study had to complete information on whether they require resources that are not related to medical care, although they were not asked to quantify them. The cost associated with VTE episode was calculated by multiplying the number of units of resource used by their respective unit cost. We obtained unit costs from the Spanish eHealth database costs (*eSalud*) [14]. Anticancer cost therapy was not contemplated, contrary to the cost of LMWH that we procured from the official Catalogue of Medicines from Official College of Pharmacists (Bot Plus) [15] considering the ex-factory price (EFP) with its respective deduction based on Royal Decree-law (RD 8/2010). All costs were expressed in €2018.

Absolute and relative frequencies were calculated for qualitative variables and statistics of central tendency (mean, median) dispersion, (standard deviation [SD], interquartile range [IQR]) or position (first and third quartile) in quantitative variables. The 95% confidence interval (CI) of the total average cost was also calculated. Data management and analysis were performed using the statistical program R 3.4.3 version (30 November 2017).

Ethics approval and consent to participate: Andalusia Biomedical Research Ethics Coordination Committee (CCEIBA) (February 2017).

## 3. Results

A total of 47 patients with LC were recruited. At the beginning of the study, the mean age was 65.4 years and 31 (66.0%) were male. Twenty-six (55.3%) had pulmonary embolism and 21 (44.7%) deep vein thrombosis. Overall, 37 (78.7%) patients had known metastases and the ECOG performance status was 0 or 1 in 70.2% of the patients. Nearly 48.9% of patients had chemotherapy as anticancer treatment. Table 1 provides the sociodemographic and clinical data.

In relation to the LMHW treatment, 53.2% of the patients were receiving enoxaparin (76.0% twice a day, 24.0% once a day), followed by tinzaparin (31.9%) and bemiparin (12.8%); for one patient the type of LMWH was not recorded (2.1%) (Table 1). Direct healthcare resource consumption and associated costs per patient in the follow-up period are detailed in Table 2. Twenty-three patients (48.9%) needed hospital admissions due to thromboembolic episodes, with an average cost (±SD) of €7207.3 (±13,996.9). The mean length of stay for hospitalizations was 9.5 days (IQR: [5.5–24.0]). Pulmonary embolism and deep vein thrombosis were the two principal causes for these hospitalizations, in 65.2%, and 21.7% of the patients, respectively. Other reasons were infections and respiratory illnesses. Inpatient stays in emergency and intensive care unit (ICU) were registered in 55.3%, and 4.2%, respectively, reporting a mean of 9 and 1 visits per patient and semester. 57.4% of patients indicated that they visited healthcare professionals during the six months of follow-up period, due to their thromboembolic disease with a mean of 1 visit per patient. Internal Medicine and Primary Care doctors were the most frequent specialists visited. A minority of participants (10.6%) indicated the need to be visited by specialists at home.

Patients who need social services represented 6.4% of patients. 2.1% of patients required informal caregiver assistance and 2.1% of patients required vehicle adaptations at home or in the workplace due to VTE.

The total average cost per patient-derived for the study period (6 months) was €10,969.6 (±14,554.2). Main cost drivers were in hospital stays and LWMH treatment. More than half of healthcare costs (65.8%) were due to hospitalization and 18.6% to LWMH therapy (€2033.8 SD: 630.5). A minority percentage represented the costs associated with emergency and ICU stays, home specialist visits, and healthcare transportation.

Due to the high SD, we present the 95% Confidence Interval for the average cost, 95% CI would be (6672.60–15,219.14). This high dispersion is explained by the highest data cost on hospital admissions of three patients with extreme values. If these patients were excluded, the average cost would be 7619.69 (±6391.56) with a 95% CI of (5861.91–9615.18), so the dispersion is lowered.

## 4. Discussion

Venous thromboembolism is one of the most common and feared complications in cancer patients, given its frequency and the suffering it entails [5]. PE is present in 2–8% of the studies performed in cancer patients [16,17].

Compared to cancer patients without VTE, cancer patients with VTE have been shown to have three times as many all-cause hospitalizations, more days spent in the hospital, and a significantly higher number of outpatient visits [18]. The main cost driver in our study was also in hospital stays, especially for those patients who suffer from PE.

Early prevention, diagnosis and treatment of CAT can save lives. Therefore, it is important to determine the cost burden of CAT on society to give policy makers some areas to adopt several strategies in government spending control, related to cancer related thrombosis, in order to invest-to-save lives and money.

Pulmonary embolism represents a pooled incidence of 3.7% in lung cancer patients [19]. In COSTECAT study more than half of patients (55.3%) had PE. It shows VTE like a key factor on mortality and morbidity in cancer patients [16].

Making a clear prognosis in PE is important. Episodes classified as low risk might be eligible for interventions, such as outpatient management or early discharge, in order to reduce costs and enhance patient comfort without compromising safety. Different studies have identified several prognostic factors for cancer-associated symptomatic PE, the most decisive among them being the presence of metastasis, immobilization, low weight, or altered vital signs [17,20].

Other studies have observed that cancer patients with VTE also have a high frequency of hospital readmission (5–17%), most of which occur within a couple of months of the initial hospitalization [21,22]. Patients with recurrent VTE had higher all-cause and VTE-related healthcare utilization (HRU) and costs compared to those without recurrence (recurrent VTE $39,641 versus without recurrence $10,499) and three times more VTE-related hospitalizations. In our study, no recurrences of VTE were observed during the six months follow-up period but three of the patients who started the study with recurrence (no first episode of VTE) presented a higher cost than patients who initiated with the first episode of VTE (€30,033.3 versus €9607.9, respectively).

Our study shows how the use of healthcare resources is an important factor in the CAT management. The direct cost of CAT is €10,969.6 per patient and semester. Lyman et al. reported an average cost per hospitalization of $37,352 for a patient and year with VTE in US medical centres, whereas the cost per patient without VTE was $19,994 ($2015). This work showed more mortality in patients with CAT and indicated a high number of clinical comorbidities and infectious complications as two high-risk factors for VTE [4]. A Swedish evaluation in patients with several cancer types and VTE reported a total cost of management of the disease for 2 years, of €14 million (€9400 per patient), almost 90% of total cost related to inpatient care [23]. In our study, we only reported the cost per hospitalization in patients with VTE and cancer, patients without VTE were excluded; thus, we cannot observe differences.

In the existing literature, there is limited information on the healthcare resource use and costs associated with LMWH in patients with cancer [24]. In a systematic review using the methodology developed by the Centre for Reviews and Dissemination (CRD) to search, retrieve, appraise and synthesize findings from a range of selected studies and this is in compliance with the Preferred Reporting Items for Systematic Reviews and Meta-Analyses (PRISMA) statement, the annual average total cost for cancer patients with VTE was found to be almost 50% higher than that of cancer patients without VTE. Inpatient care costs accounted for more than 60% of total cost. The existing evidence assessed in the present review demonstrated the significant health and economic consequences of cancer related VTE, which make a strong case for the importance of its proper and efficient prevention and management [10].

Enoxaparin was used twice daily in a significant number of patients. This pattern possibly reflects the risk of recurrence perceived by oncologists in lung cancer patients with VTE [25,26]. In our study, LMWH treatment represented the highest cost behind hospitalization (€2033.84 SD:630.47). Therefore, choosing optimal treatment option with LMWH could be considered as an optimal strategy to significantly reduce the direct cost in cancer associated thrombosis.

On the other hand, more than half of the patients did not require hospital admission in our sample of patients with lung cancer. However, we cannot rule out that PE symptomatology and the baseline ECOG may be a cause of the low admission [17]. We also cannot rule out either the confidence among oncologists in the safety of the use of LMWH in ambulatory settings as an important factor influencing on it. The cost of the disease would have been much higher if all patients with thromboembolic disease had been admitted.

The decision to admit PE patients in hospital is based on some prognostic scales in the general population, such as Pulmonary Embolism Severity Index (PESI) (or simplified version, sPESI) to predict 30-day mortality based on easily determinable clinical findings (age, cancer history, history of chronic cardiovascular disease, heart rate ≥110, systolic blood pressure <100 mm Hg, oxygen Saturation <90%). Recently, the Epiphany index was published. This is a decision tree model to stratify any cancer patient with PE according to the risk of serious complications within 15 days. This index can have potential implications for clinical management and cost. These decision-making rules are based on the combination of altered vital signs and some characteristics typical in cancer patients [17,20].

Our study could be interpreted as being underpowered to make definitive conclusions about the VTE related cost of patients with lung cancer. The small sample and the low recurrence event rate in our study population allow conclusions to be drawn only for patients without recurrence of VTE. However, given the limited data about the economic impact of VTE among cancer patients, this study fills an important gap in the current knowledge regarding the economic burden of VTE among lung cancer patients. Another potential weakness is that some data were registered retrospectively. Although, we cannot rule out that some information could be missed, we consider that this bias was reduced by the fact that all patients were interviewed to record information about economic resources used. In addition, the comparison with other studies [22] reinforces our conclusions. Finally, there has been a significant percentage of patients who did not require hospitals admission. Our study has not recorded the severity of symptoms or other variables that justify it. The fact that in several Spanish hospitals VTE is treated on an outpatient basis and that a significant percentage of cases may have been diagnosed incidentally could be a possible explanation.

## 5. Conclusions

Venous thromboembolic disease is associated with a high burden of cancer patients. A large part of the cost can be attributed to hospitalizations. Therapeutic strategies that can reduce hospital stay will result in cost savings.

## Figures and Tables

**Table 1:** Sociodemographic and clinical features of the patients.

Features	*N* = 47
Gender (male), number patients (%)	31 (66.0)
Age, mean ± SD	65.4 ± 9.5
Weight (Kg) mean ± SD	72.0 ± 15.5
Height (cm) mean ± SD	167.7 ± 8.5
Smoking, number patients (%)	20 (42.6)
Patients who do physical activity, number patients (%)	23 (48.9)
Patients with central venous catheter (%)	8 (17.0)
TNM staging system, number patients (%)	
Stage IIB	4 (8.5)
Stage IIIA	2 (4.3)
Stage IIIB	5 (10.6)
Stage IV	35 (74.5)
Unknown	1 (2.1)
ECOG performance status, number patients (%)	
ECOG 0	6 (12.8)
ECOG 1	27 (57.4)
ECOG 2	6 (12.8)
ECOG 3	5 (10.6)
ECOG 4	2 (4.3)
Unknown	1 (2.1)
Cancer stage, number patients (%)	
Locally	4 (8.5)
Locally advanced	6 (12.8)
Metastatic	37 (78.7)
Chronic comorbidities, number patients (%) *	
Stroke	4 (8.5)
Prior VTE	5 (10.6)
Obesity	3 (6.4)
Chronic venous insufficiency	3 (6.4)
Congestive heart failure (III or IV stage)	2 (4.3)
Myocardial infarction	1 (2.1)
Decompensated COPD	7 (14.9)
Infection (acute/severe)	10 (21.3)/6 (12.8)
Bleeding history	6 (12.8)
Renal insufficiency	4 (8.51)
Thrombocytopenia	6 (12.8)
Cancer treatment in VTE episode, number patients (%)	
No treatment	10 (21.3)
Chemotherapy	23 (48.9)
Targeted therapy	6 (12.8)
Immunotherapy	4 (8.5)
Palliative care	3 (6.4)
Radiotherapy	1 (2.10)
Other	4 (8.5)
LMWH treatment, number patients (%)	
Bemiparin	6 (12.8)
Enoxaparin	25 (53.2)
Tinzaparin	15 (31.9)
Unknown	1 (2.1)

* Some patients reported more than one chronic complication; COPD: Chronic Obstructive Pulmonary Disease; ECOG: Eastern Cooperative Oncology Group; LMWH: Low-molecular-weight heparin SD: Standard deviation; TNM: Tumour Node Metastasis; VTE: Venous Thromboembolism.

**Table 2:** Direct healthcare resource consumption and average cost per patient per semester.

Direct Healthcare Resource	*N* = 47 (%)	Average Cost Per Patient (€) (SD)	% Total Cost
Hospital stay *	23 (48.9)	7207.3 (13,996.9)	65.8
ICU stay **	2 (4.2)	189.9 (1000.1)	1.8
Emergency room visits	26 (55.3)	126.4 (160.1)	1.2
Hospital specialist visits	27 (57.4)	329.4 (654.3)	3.0
Laboratory tests	38 (80.9)	399.6 (567.0)	3.7
Imagine tests	38 (80.9)	601.3 (896.8)	5.5
LMWH treatment	47 *** (100)	2033.8 (630.5)	18.6
bemiparin	6 (12.8)	2613.4 (1052.6)	
enoxaparin	25 (53.2)	2173.9 (527.0)	
tinzaparin	15 (31.9)	1568.7 (153.3)	
Healthcare transportation	12 (25.5)	15.5 (30.6)	0.2
Home specialist visits	5 (10.6)	42.8 (200.2)	0.4
Out-patient rehabilitation	0 (0)	0 (0)	0
**Total Cost**	47 (100)	10,969.6 (14,554.2)	100

ICU: Intensive Care Unit; LMWH: Low Molecular Weight Heparins; * three patients present extreme values at hospital days stay compared with the other 20 patients, this explains the high dispersion in hospital stay cost. Average cost per patient (€) (SD) of hospital stay cost in 23 in-patients: 13,549.70 (16,910.94); ** only two patients with ICU stay; one with a total cost of stay of € 2479.4 and another with € 6446.4, this explains a very low average per total patient and a high dispersion. Average cost per patient (€) (SD) in n = 2 ICU patients 4462.92 (2805.12); *** one patient: treatment unknown.

## Data Availability

The data that support the findings of this study are available from Fundación Española de Farmacia Hospitalaria (FEFH) but restrictions apply to the availability of these data, which were used under license for the current study, and so are not publicly available. Data are however available from the authors upon reasonable request and with permission of Fundación Española de Farmacia Hospitalaria (FEFH).

## Abbreviations

CAT	Cancer Associated Venous Thromboembolism
DVT	Deep Vein Thrombosis
ICU	Intensive Care Unit
IQR	Interquartile Range
LC	Lung Cancer
LMWH	Low Molecular Weight Heparins
PE	Pulmonary Embolism
SD	Standard Deviation [IQR])
VTE	Venous Thromboembolism
